# Crisis response strategies and entrepreneurial orientation of SMEs: A configurational analysis on performance impacts

**DOI:** 10.1007/s11365-023-00847-4

**Published:** 2023-03-22

**Authors:** Kaisu Puumalainen, Helena Sjögrén, Juha Soininen, Pasi Syrjä, Sascha Kraus

**Affiliations:** 1 School of Business, LUT-University, Lappeenranta, Finland; 2 Business School, University of Eastern Finland, Joensuu, Finland; 3 Faculty of Economics & Management, Free University of Bozen-Bolzano, Bolzano, Italy; 4 Department of Business Management, University of Johannesburg, Johannesburg, South Africa

**Keywords:** Entrepreneurial orientation, Pivoting, Crisis management, SMEs, fsQCA, COVID-19

## Abstract

This study attempts to identify the roles of different coping strategies (pivoting and persevering) and entrepreneurial orientation that could interact and affect SMEs’ financial performance in the context of a sudden and pervasive external crisis. For this purpose, we applied asymmetric configurational theorizing and methods. The fuzzy-set qualitative comparative analysis was applied to a dataset combining survey results and financial reports of 201 Finnish SMEs. Our analysis showed that, as a response to the COVID-19 crisis, it was more common to apply pivoting than the perseverance strategy. Entrepreneurial orientation was positively related to pivoting, growth, and subjective performance but not related to profitability. Firms that had faced more problems tended to apply the perseverance strategy, and their financial performance was weaker. We also found combinations of factors that led to poor performance as non-entrepreneurial firms that faced major problems consistently performed poorly despite the coping strategies employed. In conclusion, we argue that different types of coping strategies are powerful and effective in different intensities of crises experienced by SMEs, but it is critical to match the correct coping strategy with the firm’s strategic orientation.

## Introduction

Wars, pandemics, and natural disasters are examples of sudden external crises that can threaten firms’ survival locally and globally. The COVID-19 pandemic is such an unexpected crisis, which has impacted firms across all sectors and industries on a global scale (Sharma et al., 2022). It is typical for crises that they come unexpectedly, and their duration cannot be predicted (e.g. what will happen in China regarding COVID-19 is still completely uncertain). Meanwhile, the war in Ukraine has affected firms in many ways, e.g. forced them to abruptly exit from Russian markets, caused the prices of electricity and raw materials to increase sharply, and compromised the availability of production inputs. Other crises in the near future could be caused by inflation and rising interest rates in Europe. The emergence of these crises raises important questions about how firms can respond effectively to external threats (Bouncken et al., 2022). In the case of COVID-19, restrictions imposed by governments have challenged businesses and even locked down businesses, threatening their survival (Emami et al., 2021). However, companies in different industries were affected differently by the crisis. Klyver and Nielsen (2021) classified these into three categories: *crisis exploiters*, *crisis immunes*, and *crisis victims*. In general, crises and disasters make it particularly difficult for SMEs to access resources and create liquidity problems (Eggers, 2020; Kraus et al., 2013). However, Belitski et al. (2021) pointed out that small businesses are very resilient during the crisis, as they focused on studying how the COVID-19 pandemic affects entrepreneurship and SMEs around the world. Resilient entrepreneurs have several individual characteristics (e.g. optimism, proactiveness, perseverance, motivation, flexibility, and self-efficacy) that enable them to respond more effectively to a crisis (Portuguez Castro & Gómez Zermeño, 2021). E.g. self-efficacy of the entrepreneur and the level of stress she/he experiences can affect the company's coping strategy (Meyer et al., 2022).

Literature on resilience in business and management is increasing (Hillmann, 2021) and previous studies (Bressan et al., 2021) have highlighted key actions by SMEs to strengthen their resilience to crises. Basically, a firm can respond to an external threat in two ways, either by *defending* or using an *offensive* approach (Manolova et al., 2020). Defensive actions include deferring investments, reducing expenses, reducing labour costs, and renegotiating agreements. Instead, when dealing with offensive options, SMEs focus on opportunities for innovation (Kuckertz & Brändle, 2021). Crises can also create new opportunities (Klyver & Nielsen, 2021), and innovative entrepreneurs can develop new products and services or introduce new technological ways to perform their businesses (Clauss et al., 2021). Ebersberger and Kuckertz (2021) investigated what type of innovator was the quickest to react to the opportunities and challenges followed by the COVID-19 crisis. They demonstrated that innovative startups are the quickest to react to the changing business environment. They highlight that crises offer new opportunities for innovative startups, and they respond faster than established organizations. Whereas new firms are still seeking opportunities and have not locked themselves into a particular model, mature firms have a history that narrows their search options and may result in path dependency (Schreyögg & Sydow, 2011). Mature firms make strategic changes when they perceive a performance gap between their target and expected performance (Levitt & March, 1988). Strategic change can also be seen as a result of an external crisis. The difficulty of forecasting the duration of the crisis has motivated some firms to consider changing their products, services, customers, or markets (Morgan et al., 2020). This kind of business model innovation can be a tactical approach for SMEs to face the crisis (Clauss et al., 2021). In this study, we used the commonly used term pivoting for this kind of innovative strategic reorientation. Successful business model pivots, particularly in response to serious economic shocks, must simultaneously reduce risk and create opportunities (Manolova, 2020; McGrath & Macmillan, 2009). Naturally, entrepreneurs’ response to the crisis depends largely on the resources available during the crisis (Kuckertz & Brändle, 2021). If resources are already scarce at the beginning of a crisis, the options for responding to the crisis will be more limited. Retrenchment has been considered the most common strategy that SMEs select in response to crises (Bruton et al., 2003). In particular, at the beginning of a crisis, this cost-cutting strategy may be the only possible short-term action (Wentzel et al., 2020). Also, persevering can be an easy strategic option for SMEs to survive. It only focuses on maintaining the company’s existing operations (Kraus et al., 2020) and mitigating the adverse effects of the crisis by reorganizing activities, e.g. loans, contracts, and terms negotiations with suppliers and stakeholders (Klyver & Nielsen, 2021). Like previous studies (e.g. Klyver & Nielsen, 2021; Kraus et al., 2020), we followed Wentzel et al.’s (2020) conceptualization of crisis coping strategies, focused on continuing firms and accordingly left out the exit strategy, and replaced innovating with pivoting, leaving the following three: persevering, retrenchment, and pivoting.

In addition to various coping strategies and individual determinants of crisis resilience, the role of organizational and strategic routines is important also in SMEs. Dejardin et al. (2022) found that dynamic capabilities by which firms obtain new configurations of resources in keeping with how markets create, evolve or die is a key determinant of SME performance in the COVID-19 crisis. This study will contribute to the existing knowledge by focusing on another organizational and strategic routine, entrepreneurial orientation (EO). Several scholars have established a link between EO and firm survival (e.g. Eggers, 2020; Nofiani et al., 2021; Sufyan et al., 2021). Firms with EO can change their operations more efficiently in times of crisis, which increases the company’s overall performance (Lekmat & Chelliah, 2011). Overall, several studies underline the importance of EO when firms face harsh external economic threats. The results of Covin and Slevin (1989) showed that more entrepreneurial firms tend to generally perform better in hostile environments than less entrepreneurial firms. Soininen et al. (2012a) showed that different dimensions of EO influence how Finnish small firms face sudden recession as higher levels of innovativeness and proactiveness have a positive effect on a firm’s financial performance; on the other hand, risk-taking has an opposite effect as it harms a firm’s financial performance. The results of Beliaeva et al. (2020) revealed that firms are able to find the best opportunities present during an economic crisis with high levels of EO. Hence, all these findings underline the importance of EO when firms face crises. Elements of EO (innovativeness, proactivity, and risk-taking) can therefore be said to constitute *preconditions* of resilience (Kuckertz & Brändle, 2021). Thus, entrepreneurially oriented firms also outperform their less entrepreneurial counterparts (and especially) in challenging circumstances, but it is less clear whether and how EO interacts with crisis management strategies. As Linton and Kask (2017) argued, neither EO nor the strategy of choice might be sufficient to explain firm performance in isolation from one another, but combining a firm’s EO posture correctly with its competitive strategy might affect performance positively. Moreover, Linton and Kask (2017) underlined that there is a need for further research to examine EO in configurations with other aspects, such as strategy. Similarly, Covin et al. (2020) pointed out that the relationship between EO and performance might be more complex than previously assumed, and hence studies that focus on the interplay between EO postures and firm-level strategies are necessary. As a strategic orientation like EO is hard/impossible to change, coping strategies should be aligned with it. We address the following research question: *How can SMEs employ different configurations of EO and crisis coping strategies to achieve high performance (or to avoid poor performance) amid an external crisis?* To this end, we applied asymmetric configurational theorizing and methods. Fuzzy-set qualitative comparative analysis (fsQCA) was applied to a dataset combining survey results and financial reports of 201 Finnish SMEs. The asymmetric configurational approach (see e.g., Iannacci & Kraus, 2022), to our knowledge, has not been applied in the context of SMEs coping with crises. We believe that in crisis circumstances, avoiding poor performance becomes relatively more important for SMEs than achieving good performance; thus, it is important that any causal asymmetries can be revealed.

This study contributes to the EO literature in two ways. First, we show the ‘dark side of EO’, in other words, how a high level of EO can also result in poor performance. Second, we propose a model for how EO can support the application of coping strategies and the effect of these combinations on performance.

The paper further contributes to the literature on strategic and crisis management of SMEs during external crises and shows how different coping strategies are effective in different intensities of the crisis experienced by the firms. We show evidence that firms trying to apply coping strategies are not always successful. We highlight the difficulty of finding the balance in a crisis, especially when firms tend to use both persevering and pivoting strategies simultaneously. We contribute to pivoting research by confirming the role of external problems as a major reason to pivot and show empirical relations between pivoting and a firm´s performance, especially in the context of mature SMEs. This paper also complements the literature on the impacts of COVID-19 by using a combination of survey and archival data and is one of the first such quantitative studies in the management field.

## Literature review and proposition

### Coping strategies of SMEs in crises

Entrepreneurship is a process that inherently includes uncertainty (McMullen & Dimov, 2013), but coping with sudden external threats also poses challenges for entrepreneurial firms. For instance, the COVID-19 pandemic threatened firms’ survival worldwide. In many industries, restrictions imposed by governments have challenged businesses and even locked down nonessential businesses. Due to the uncertainty already associated with entrepreneurship, entrepreneurs are likely to confront unexpected events that require them to consider whether to persevere with their original ideas or pivot from them (Kirtley & O´Mahony, 2023). Berends et al. (2021) highlighted that when considering the question of persevering or pivoting, entrepreneurs must reconsider the relational and temporal commitments associated with their business, balancing the question of changing strategic orientation or not.

Sometimes, an external shock may require immediate actions before SMEs can even consider the question of whether to persevere or pivot. As a direct short-run response to a crisis, reducing costs has an incredibly positive effect on maintaining liquidity and providing a sustainable foundation for recovery (Pearce & Robbins, 1994). This retrenchment strategy (Wentzel et al., 2020) is therefore based on cost-cutting and is used mostly in situations where an SME lacks resources (Giones et al., 2020). According to Wentzel et al. (2020), retrenchment can be the first and maybe the most common strategy in recovering from a crisis, but that alone may not be enough. They highlighted that retrenchment could also have a negative impact on the company’s performance. Reductions in costs, assets, and products narrow the firm’s business opportunities.

Kirtley and O´Mahony (2023) showed that after considering strategic change, entrepreneurs most often refused to implement it. They found their results to be consistent with previous research that showed that entrepreneurs can be passionate to the point of persistence (Cardon et al., 2009), can identify strongly with the products they develop (Elsbach & Flynn, 2013), or can resist change (Grimes, 2018). Persevering focuses on maintaining the company’s existing operations (Kraus et al., 2020). Therefore, it could be assumed that persistence is an easier strategic option for SMEs to survive. When choosing preserving as a survival strategy, SMEs need to reinforce the original business idea through the maintenance and extension of their earlier choices of technologies, offerings, customers, or partners (Berends et al., 2021). In addition, close to the concept of persevering is the concept of resilience, which refers not only to restoring the business prevailing before the crisis but also to developing it to perform at least as well under the new conditions after the crisis (Hamel & Välikangas, 2003). In the event of a crisis in which firms are confronted with changed circumstances on a day-to-day basis, persevering can be a surprisingly effective strategic choice (Pacheco-de-Almeida, 2010; Stieglitz et al., 2016; Wentzel et al., 2020). According to Wentzel et al. (2020), persevering can help a company survive in the medium term but is not useful in long-term crises. Perseverance is largely based on the limited availability of resources. As the crisis continues for a longer period, these resources could decrease rapidly. In contrast, strategic renewal related to pivoting requires the commitment of resources.

When SMEs choose pivoting as a coping strategy, they need to change a business idea or business model and make changes in technologies, offerings, or relationships with customers or business partners (Ries, 2011, 2017). The concept of pivot is defined and used in different ways by different researchers, but in general, this choice of strategy is always accompanied by major behavioural changes. A study by Ries (2011) defined pivot as strategy change without changing a vision. Similarly, Ester and Maas (2016) defined pivot as an effective entrepreneurial practice. Some researchers see that pivoting happens when resource-constrained enterprises come to view their current business model and life cycle as unsustainable and decide to transform themselves in an effort to survive and grow (Grimes, 2018; Kunisch et al., 2017). On the other hand, pivot is seen as a change in an enterprise’s strategy through a reallocation or restructuring of activities, resources, and attention (Kirtley & O´Mahony, 2023). Simultaneously, not every action by an entrepreneur can be seen as a meaningful pivot since action has to have fundamental consequences (Ries, 2011, 2017).

Whereas new firms are still seeking opportunities and have not locked themselves into a particular model, mature firms have a history that narrows their search options and may result in path dependency (Schreyögg & Sydow, 2011). Current theories suggest that mature firms especially make a strategic change when they perceive a performance gap between their target and expected performance (Levitt & March, 1988). Pivoting is also seen as a consequence of exogenous shocks (Morgan et al., 2020). The concept pivot is also seen as different from strategic change. Leatherbee and Katila (2017) argue that pivot differs from strategic change and that pivoting is a quick cognitive change in the opportunity logic or strategi logic of a business model. Furthermore, Kirtley and O´Mahony (2023) see that pivoting is not a single, sweeping move but rather the end of a process of strategic recalibrations triggered by challenges and new possibilities. In some cases, enterprises turn to pivot through customer dissatisfactions, and this leads to iterations of the initial offering and changing of the business model (Andries & Debackere, 2006). Pivoting may be a traumatic event for the firm, and it can lead to business failure (Molly et al., 2010). Shepherd (2020) argues that we should look for pivots by everyday entrepreneurs rather than by heroic entrepreneurs. Overall, it is almost impossible for an entrepreneur to know in advance which strategic choice will lead firms to survive a crisis and recover after the shock (Pearson & Clair, 1998), and strategy choices also largely depend on available resources.

### Entrepreneurial orientation in a crisis context

EO has arisen as one of the key constructs within strategic management and entrepreneurship literature, as an extensive number of studies have focused on the concept during the past four decades. According to Wales et al. (2021), EO characterizes an organizational orientation towards new entry and value creation, as it captures the entrepreneurial decisions and actions actors use to achieve competitive advantage. EO is a multidimensional concept. For instance, Wiklund (1999) and Miller and Le Breton-Miller (2011) pointed out that there is a consensus among most researchers that EO is, based on the model created by Miller (1983), a combination of three dimensions: innovativeness, proactiveness, and risk-taking.

*Innovativeness* is, according to Lumpkin and Dess (1996), pursuing and giving support to novelty, creative processes, and the development of new ideas through experimentation that may result in new products, services, or technological processes. The second dimension is *proactiveness,* which can be described as an opportunity-seeking, forward-looking perspective characterized by the introduction of new services and products ahead of the competition and acting in anticipation of future demand (Semrau et al., 2016). Kraus et al. (2012) conclude that proactiveness concerns the importance of the initiative in the entrepreneurial process and, for instance, Venkatraman (1989) defines proactiveness as processes that are aimed at looking for new opportunities that may or may not be related to the present line of operations, introduction of new products and brands ahead of the competition, and strategically eliminating operations that are in the mature or declining stages of the life cycle. Lumpkin and Dess (1996) argued that this anticipation of changes in future demand could give a competitive advantage to a proactive firm compared with its less proactive competitors. The third dimension of EO is *risk-taking*, which can be defined as ‘the degree to which managers are willing to make large and risky resource commitments’ (Miller & Friesen, 1978, p. 923). When a firm is behaving entrepreneurially, for instance, investing a substantial amount of resources in a venture prone to failure, this may cause uncertainty, but as Kraus (2013) points out within the entrepreneurship framework, risk-taking is not reckless but rather controlled and calculated.

The relationship between EO and business performance has been studied intensively in recent decades (see, for instance, Wales et al., 2021), leading to a situation where EO has become a valuable and generally acknowledged determinant of firm performance (Hernández-Perlines et al., 2021). Dozens of empirical studies have indicated that firms with higher levels of EO have outperformed firms with lower levels of EO (e.g. Donbesuur et al., 2020; Hernández-Perlines et al., 2021; Kallmuenzer et al., 2018; Kraus et al., 2012; Soininen et al., 2012b; Wiklund & Shepherd, 2003). This positive link between EO and business success has been found to exist across different types of firms (Baker & Sinkula, 2009; Gupta & Gupta, 2015), industries (Buli, 2017; Kraus, 2013), and countries (Javalgi & Todd, 2011; Razak, 2011).

Beliaeva et al. (2020) state that there is a growing interest in exploring strategic orientations, their interaction, and their impact on firm performance. It is important to note that a majority of studies have been conducted within stable economic environments. Even though this is true, there are also studies that have explored the role of EO under unstable market conditions, for instance, during a financial crisis or a global pandemic. Covin and Slevin (1989) were one of the first to study the relationship between an entrepreneurial strategic posture (i.e. EO) and firms’ financial performance of small manufacturing firms in hostile and benign environments. The results of Covin and Slevin (1989) show that more entrepreneurial firms tend to generally perform better in hostile environments than less entrepreneurial firms. Krishnan et al. (2022) also point out that EO has an essential role in managing a crisis/disaster. The latest exogenous shock causing major disruptions to economic systems is the global COVID-19 pandemic, and a few studies have already explored the role of EO under this pandemic. Li et al. (2021) focus on this issue by exploring how EO influences the growth of manufacturing firms during the COVID-19 pandemic. Using a sample of some 700 Ghanaian SMEs, Li et al. (2021) showed a significant positive direct relationship between EO and firm growth. Instead of the EO–performance relationship, Zighan et al. (2021) studied how EO impacts SMEs’ resilience (i.e. an organization’s ability to recover from a crisis.) during the COVID-19 pandemic in Jordan. The study showed that EO increases the tolerance for uncertainty regardless of resource limitations, especially risk-taking and innovativeness, which are essential elements to help companies quickly adapt to change. Also, the study by Meyer et al. (2021) highlighted the role of entrepreneurial posture under special circumstances, as they stated that entrepreneurship helps societies pull through crisis and assists societies in surviving the social and economic disruptions of the pandemic; in other words, the crisis creates entrepreneurial opportunities. Entrepreneurial businesses identify social needs and develop novel solutions to those needs. Such businesses produce entrepreneurial innovations that cover innovations in business models and technologies and that shape the new normal for post-COVID societies. On the individual level of analysis, a survey by Salmony et al. (2022) studied how the entrepreneur’s personality affects their perceptions about the extent of impact that COVID-19 had on their businesses. The study found that risk-taking propensity was higher among those who experienced stronger impact of COVID-19 (either positively or negatively) on their business. Interestingly, innovativeness was not significantly related to perceived impact on business.

To sum up, the research on EO has shown that EO is a firm-level strategic orientation that has a positive main effect on firm performance, also in the context of challenging environments like global crises. However, it is less understood how this overall and rather generic disposition is linked to the concrete actions that firms take as a response to an external crisis.

### Possible recipes for coping with the crisis

Our theoretical framework aimed to identify the roles of different coping strategies and EO that could interact and affect SMEs’ financial performance in the context of a sudden and pervasive external crisis, such as the COVID-19 pandemic.

First, the crisis hits SMEs in different ways and to different extents; e.g. the requirements for social distancing and lockdowns would immediately cut the sales revenues of restaurants and organizers of public events, while the demand for certain products like handicrafts and gardening-related goods increased. Therefore, the amount and severity of the problems faced by the firms would not only impact the financial performance directly but probably also interact with other factors. We can expect that different types of strategies are effective in coping with minor problems vis-à-vis major ones. For example, the perseverance strategy can work best in the context of minor, short-term problems, but long-lasting severe crises may require more innovative strategies, such as pivoting (Berends et al., 2021; Covin & Slevin, 1989).

Second, while it has been established in previous research that entrepreneurially oriented firms outperform their less entrepreneurial counterparts (especially) in challenging circumstances (Zighan et al., 2021), it is less clear whether and how EO interacts with the crisis coping strategies. We expect that more entrepreneurially oriented SMEs would be more likely to pivot and be more effective in pivoting than firms with lower EO (Kirtley & O’Mahony, 2023). On the other hand, less entrepreneurial firms are less willing to take risks and may therefore be more effective in finding and exploiting the perseverance strategies that enable them to overcome the crisis.

Third, the coping strategies could interact with each other in their effects on firm performance. A pivoting strategy based on finding new revenues might be hard to combine with efforts to persevere the status quo or to achieve cost-savings. On the other hand, the use of several coping strategies at the same time could be a way to secure financial performance (Pearson & Clair, 1998).

**Fig. 1:**
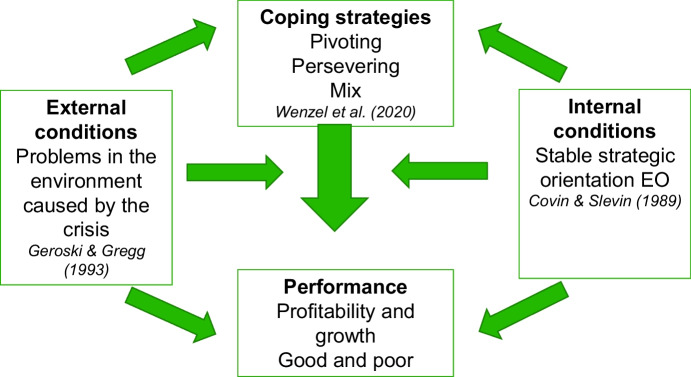
Proposed configurational model

Therefore, we summarize our theoretical reasoning in the following proposition (see also Fig. 1):

*In the context of an external crisis, such as the COVID-19 pandemic, the effectiveness of a firm’s coping strategies depends on the severity of the problems faced and the firm’s EO.*

## Methodology

### Fuzzy-set qualitative comparative analysis

In our analysis, we adopted the QCA approach (Kraus et al., 2018; Kumar et al., 2022; Longest & Vaisey, 2008; Ragin, 1987, 2000). Longest and Vaisey (2008, p. 79) noted that instead of estimating the net effects of single variables, ‘QCA employs Boolean logic to examine the relationship between an outcome and all binary combinations of multiple predictors’. They further suggested that this approach is appropriate for testing ‘social and behavioral theories that posit combinations of variables working in highly contingent ways’. QCA differs from traditional explanatory models because it supports the issue of equifinality (Munoz & Cohen, 2017). In our case, this would mean that a certain level of firm performance can be achieved through several different configurations of EO, COVID-induced problems, and coping strategies. Another strength of QCA is its ability to capture causal asymmetries (Fiss, 2011), releasing the (often hidden) assumption that the causal conditions leading to superior performance are the opposite of those leading to inferior performance. QCA can also be utilized when the sample size is small or moderate (with the latter applying to our case), as long as overfitting can be avoided by careful selection of a limited number of causal conditions. Traditional multivariate analysis methods usually require 5–10 times as many cases as there are variables in the model, and with the main and interaction effects of several independent constructs, each measured by multi-item scales, the required sample size would usually exceed 200 cases. Furthermore, sample representativeness is not as critical as in traditional linear models because QCA does not rely on assumptions of normal distributions. In our analysis, we employed fsQCA with the ‘fuzzy’ procedure in Stata SE 17 software.

### Sampling and data collection

The empirical data used to study the performance implications of EO and crisis coping strategies were collected from a sample of 201 Finnish SMEs employing between 10 and 99 people from six industry categories: manufacturing, construction, trade, logistics, hospitality, and health/social services. Finland was selected as a context for this study because of access to high quality data on SMEs, particularly the ability to combine survey responses with financial register data. However, regarding our main constructs of interests (effects of crisis on SMEs, crisis response strategies, EO and financial performance) there should be no major differences between Finland and other similar developed countries. The six industries were selected based on two criteria: (1) the included sectors should substantially large, i.e. include a lot of companies and (2) because it was assumed that the COVID-19 pandemic might have different effects on the selected industries. E.g. manufacturing and construction are large industry sectors, but were not expected to suffer very much from the pandemic. On the other hand, logistics and hospitality were assumed to take a severe hit because of the COVID-19 restrictions. Health services was included because the crisis in our study was a pandemic threatening people’s health.

The survey was targeted at owner-managers of the SMEs, and the questionnaire included items concerning the impacts of COVID-19, the firm’s strategic orientations and performance, and the entrepreneur’s decision-making style and background, among others. Some of the items in the questionnaire were negatively worded to avoid agreement bias and to account for potentially extreme response styles (see Baumgartner & Steenkamp, 2001). Before data collection, the survey questionnaire was piloted with three SME managers.

The survey was executed from November 2020 to December 2020 via computer-aided telephone interviews, and a large market research company was employed for data collection*.* A stratified sample contained 13,934 companies from the Bisnode company register*,* which covers all Finnish enterprises, and 8,442 firms (61% of the sample) were contacted. Out of the contacted enterprises, 201 responded to the survey, thus resulting in a modest response rate of 2.38%. This can be partly because the survey covered an extensive range of questions, and the interviews took an average of 30 min. Achieving higher response rates has been a challenge not only in Finland (e.g. Autio et al., 2000) but also in surveys targeted at SMEs elsewhere (Bartholomew & Smith, 2006; Newby et al., 2003).

Non-response bias was evaluated by comparing the background characteristics of respondents and non-respondents (see Table 1). As the industry and firm size distributions of respondents compared to non-respondents were nearly identical and none of the chi-square tests were even close to statistical significance, the sample was found to be representative despite the low response rate.

**Table 1:** Descriptive information about the sample and non-response analysis

Industry	Respondents (*N*)	Respondents (*%*)	Non-respondents (*N*)	Non-respondents (%)	Comparison tests
Manufacturing	46	22.9	1967	23.3	Chi-Square = 0.11, df = 5, *p* = 0.9998
Construction	48	23.9	2035	24.2	
Trade	46	22.9	1940	23.0	
Logistics	22	11.0	925	10.9	
Hospitality	20	10.0	850	10	
Health/social services	19	9.5	725	8.6	
Total	201	100	8442	100	
Employees	Respondents (*N*)	Respondents (%)	Non-respondents (*N*)	Non-respondents (%)	Comparison tests
10–19	142	70.6	6003	71.1	Chi-Square = 0.09, df = 2, *p* = 0.9560
20–49	54	26.9	2190	26.0	
50–99	5	2.5	249	2.9	
Total	201	100	8442	100	
Sales	Respondents (*N*)	Respondents (%)	Non-respondents (*N*)	Non-respondents (%)	Comparison tests
200–399 k€	4	2.0	185	2.2	Chi-Square = 0.06, df = 3, *p* = 0.9959
400–999 k€	28	13.9	1203	14.3	
1–2 M€	74	36.8	3155	37.3	
2–10 M€	95	47.3	3899	46.2	
Total	201	100	8442	100	

To minimize the potential for common method bias, several steps were taken before and after data collection in line with the recommendations of Podsakoff et al. (2003) and Chang et al. (2010).

First, the items of outcome and condition variables were placed in different sections of the questionnaire, and negatively worded items were included to avoid any halo effects. Second, it was unlikely that the respondents would have been guided by any assumed model of relationships as the focal items were part of a larger survey covering a range of issues for SMEs beyond EO, financial performance, and pandemic-related issues. Furthermore, the purpose in this study is to identify configurations of the condition variables that are harder to visualize by the respondents than simple main effects (Chang et al., 2010, p. 179).

Third, Harman’s single-factor test was conducted by running a principal component analysis on all the focal items of the study. The results indicated that the first factor only accounted for 24.5% of the total variance and that the items of the outcome construct (subjective financial performance) clearly loaded on a factor of its own, while the items of other key constructs (EO, COVID-related problems, and the coping strategies) loaded on their respective factors.

Fourth, by including two objective performance indicators from the companies’ financial reports as a robustness test, we were able to test our proposed model with data where the outcome variables came from a different source than the condition variables (Podsakoff et al., 2003).

### Measures

#### Outcomes

To add robustness to the results and to account for potential common method bias, both subjective and objective measures of firm performance were used in the analysis. The subjective performance measure was composed as a mean of three items on a Likert scale, with a Cronbach’s alpha value of 0.691 (see Appendix 1 for the individual items). The objective measures of firm performance were return on assets (ROAs) and sales growth percentage, taken from the companies’ financial reports regarding the fiscal year 2020 and retrieved from the Amadeus database. Taken together, the measures of performance capture financial performance from the perspectives of profitability, growth, and continuity/resilience.

#### Causal conditions

##### Entrepreneurial orientation

We utilized nine items to capture the three dimensions of EO conceptualized by Miller (1983). The items are based on the work of Covin and Slevin (1990). However, the item wordings were slightly adapted to better fit the context of Finnish SMEs. A principal component analysis of the EO items (see Appendix 1, Table 6) resulted in two components together explaining 61% of the variance in the items. The first component had an eigenvalue of 4.43 and the second 1.03. In the unrotated factor solution, all items except one loaded highly on the first component. Therefore, we decided to treat EO as a unidimensional construct in the analysis, following Covin and Slevin (1989). The internal consistency of the scale was good, as Cronbach’s alpha value was 0.861.

##### COVID problems

The items for COVID-related changes in the firms’ operating environments and their response strategies were developed for the purposes of this study. However, previous studies about the impacts of financial crises on companies’ operations and their ways of coping were reviewed for possible items. In their study, Geroski and Gregg (1993) created 32 items to measure how severely firms were affected by the recession, how severely it affected various elements of their trading position, and what their major problems had been. Later, Soininen et al. (2012a) built upon the work of Geroski and Gregg (1993) and created 20 items to gauge what kind of impact the financial crisis of 2008 had on (1) sales and profitability, (2) short-term financing, (3) long-term financing, (4) personnel, (5) competitive situation, and (6) payment terms of Finnish small firms. As the items of Soininen et al. (2012a) are proved to be suitable in the context of Finnish SMEs, we utilized some of those items with the same scale in our study to investigate what kind of acute problems firms have faced related to sales and profitability, short-term financing, and payment terms. Items were assessed on a five-point Likert scale with the anchors 1 = totally disagree and 5 = totally agree.

##### Coping strategies

The questionnaire included 15 items to measure the actions taken by the companies in response to the crisis. Principal component analysis with the varimax rotation method initially resulted in three factors with eigenvalues larger than 1. However, the contribution of the third factor to the variance explained was only 8%, and thus a two-factor solution was selected (see Appendix 1, Table 7). In the solution, 10 items were loaded on the first factor, which explained 34% of the total variation and was named perseverance. The final measure was computed as an average of the 10 items, and it had a good internal consistency with a Cronbach alpha of 0.867. The five items related to innovation and efforts to gain new revenues were loaded on the second factor, which explained 16% of the total variance. These items were also averaged, and the resulting scale was named pivoting. The reliability coefficient for pivoting was 0.745.

### Measure calibration

To create sets for analysis using fsQCA, the variables first need to be ‘fuzzified’ or calibrated to range between 0 and 1 (Ragin, 2008). There are several approaches for calibration, ranging from purely theoretical and substantive knowledge-based reasoning to empirically driven calibration based on the distributional characteristics of the variables.

In our main analysis, we applied the standardized rank procedure (Longest & Vaisey, 2008), where the cases are first ranked according to the values of the original variables. The ranks are then standardized by subtracting the minimum rank and dividing the result by the difference between the maximum and minimum ranks. This results in scores between 0 and 1. The same calibration method has previously been applied in studies published in high-level business and management journals (e.g. Chen & Lin, 2019).

As the calibration method is critical to the robustness of the sets and to the subsequent analysis, we also tested for alternative methods of calibration. In the robustness checks, we used a direct calibration where we manually assigned thresholds for full non-membership, cross-over point, and full membership, respectively. For the objective performance measures, we applied theoretical thresholds as follows: The growth percentage was deemed to be fully out when sales had declined 20% or more, the cross-over point was set at the decline of 10%, and full membership when the sales had not decreased at all or even grown during the pandemic. ROA thresholds for poor, average, and good performance were 0, 5, and 10, which are commonly listed as guidelines on the interpretation of the ROA ratio (Yritystutkimus ry, 2017). The outcome subjective performance and all conditions were calibrated by applying distributional rather than theoretical thresholds because they were based on Likert-scale items, which may be subject to social desirability bias or response styles (Baumgartner & Steenkamp, 2001), resulting in distributions that do not range within the whole spectrum of the scale anchors. Following recent studies (De Crescenzo et al., 2020; Mena et al., 2022; Santos et al., 2018), we placed the thresholds for fully out, cross-over point, and fully in at the 10th,50th, and 90th percentiles of the distribution in all variables (see Appendix 2, Table 8). As the fuzzy scores at the cross-over point of maximum ambiguity would present difficulties in further analyses, we added a small constant of 0.001 to the scores in accordance with established practices (Chen & Tian, 2022; Fiss, 2011).

## Results

### Descriptives and analysis of necessity

The descriptive information of all original variables applied in the analyses is shown in Table 2. On average, the SMEs considered themselves rather entrepreneurial and evaluated their own performance to be somewhat better than their competitors’. The mean value for COVID-induced problems was only 2.3, indicating that most of the SMEs had not faced severe problems during the first year of the pandemic and did not have to cut the costs to persevere to a notable extent (mean value 2.0). The average ROAs for the year 2020 was 12%, which can be considered good profitability, but the variation between the firms was very large. The same applies to sales growth percentage in 2020 compared to 2019: the mean is 15% growth, but with a large standard deviation.

**Table 2:** Descriptive statistics and correlations

	EO	Problems	Pivoting	Perseverance	Subj.perf	ROA	Growth%
EO							
Problems	0.119*						
Pivoting	0.458***	0.186***					
Perseverance	0.089	0.786***	0.208***				
Subj.perf	0.224***	− 0.311***	0.027	− 0.278***			
ROA	− 0.039	− 0.397***	− 0.056	− 0.363***	0.237***		
Growth%	0.202**	− 0.098	0.151*	− 0.138	0.078	− 0.113	
Mean	3.422	2.301	2.791	2.008	3.667	11.966	15.192
Std. Dev	0.715	0.928	0.938	0.946	0.677	16.518	83.264
*N*	201	201	201	201	201	145	136
Cronbach alpha	0.861 (9 items)	0.860 (9 items)	0.745 (5 items)	0.867 (10 items)	0.691 (3 items)	n.a	n.a

The correlations in Table 2 indicate that EO is strongly and positively associated with the pivoting strategy, while the perseverance strategy correlates positively with the intensity of problems faced during the pandemic. Subjective performance and ROA are both negatively related to problems and perseverance efforts, while growth has a small positive correlation with pivoting and EO.

Following the suggestion of Schneider and Wagemann (2010), we begin QCA with the analysis of necessity, applying the threshold of 0.90 for consistency, as suggested in Greckhamer et al. (2018). This means that a causal condition can only be interpreted as a necessary condition if more than 90% of the cases exhibiting the condition are also exhibiting the outcome. Overall, the consistencies are rather low in Table 3, and none of our causal conditions are necessary for any of the outcomes. The lower part of Table 3 shows coverages, i.e. how many percent of cases with the outcome have the causal condition.

**Table 3:** Analysis of necessity

	Subj.perf	ROA	Growth%
Consistencies			
High EO (E)	0.724	0.649	0.690
High problems (P)	0.624	0.602	0.593
High pivoting (V)	0.683	0.658	0.647
High perseverance (C)	0.621	0.591	0.592
Coverages			
High EO (E)	0.706	0.654	0.705
High problems (P)	0.599	0.597	0.581
High pivoting (V)	0.659	0.658	0.655
High perseverance (C)	0.560	0.545	0.547

### Analysis of sufficiency – Truth tables

Following the example of Cannaerts et al. (2019), we report truth tables as a first step of the analysis of sufficiency. As we employ four causal conditions (*EO*, *Problems*, *Pivoting*, and *Perseverance*), the number of potential configurations is 2^4^ = 16. Table 4 is a truth table showing the frequencies of occurrence for each of these 16 configurations in our data set of 201 firms. The most common configuration is the one where the firm has high EO, meets a lot of problems, and tries to cope by both pivoting and perseverance strategies. This configuration (EPVC) covers 36 firms, representing 19% of all firms in the sample. One potential configuration (EPvc) of the causal conditions was not observed at all in the data. A closer look at this counterfactual (also known as logical remainder) reveals that it would represent a firm with high EO and a lot of problems but no attempt to pivot or persevere by reducing costs. This seems theoretically implausible, so the configuration was excluded from further analyses.

**Table 4:** Truth table

			Consistencies					
Config	*N*	%	Subj.perf. good	ROA good	Sales growth	Subj.perf. poor	ROA poor	Sales decline
EPVC	36	17.91	0.77	0.71	0.72	0.81	0.83	0.81
EPVc	11	5.47	0.86*	0.89*	0.85	0.81	0.84	0.84
EPvC	13	6.47	0.82	0.81	0.81	0.85	0.87*	0.86
EPvc	0	0	0.89	0.91	0.89	0.86	0.88	0.89
EpVC	4	1.99	0.90*	0.93	0.91	0.82*	0.83	0.81
EpVc	19	9.45	0.89*	0.93*	0.90*	0.72	0.76	0.75
EpvC	1	0.5	0.91	0.92	0.90	0.87	0.86	0.87
Epvc	23	11.44	0.89*	0.87*	0.87*	0.71	0.76	0.76
ePVC	11	5.47	0.78	0.78	0.75	0.89*	0.86	0.91*
ePVc	3	1.49	0.86	0.93	0.85	0.89	0.85	0.92
ePvC	21	10.45	0.77	0.79	0.75	0.87*	0.84	0.88*
ePvc	4	1.99	0.84	0.89	0.84	0.88*	0.86	0.89
epVC	5	2.49	0.88*	0.92	0.88	0.86*	0.85	0.90
epVc	12	5.97	0.87*	0.95*	0.89*	0.81	0.77	0.83
epvC	3	1.49	0.85	0.87	0.86	0.87	0.87	0.91
epvc	35	17.41	0.77	0.84	0.83	0.76	0.72	0.74
Total *N*	201		201	145	136	201	145	136

Configurations marked with * are included in the Boolean minimization

As for the other low-frequency configurations, we decided to apply a frequency threshold of three for inclusion in the analyses. When the total number of cases in a QCA analysis is relatively small, the frequency cutoff is usually one or two, but with large samples, a larger threshold should be used (Ragin, 2017, pp. 39–40). Our sample size (N = 201) is above 50 and can thus be considered rather large than small in the QCA context (Greckhamer et al., 2013). Furthermore, as the data do not have in-depth information on every individual case, a threshold above one or two can be justified. Applying the threshold of three cases retains all but seven of the original 201 cases, which meets the recommended 80% retention guideline (Greckhamer et al., 2013, p. 66). Thus, configurations EPvc, ePVc, epvC, and EpvC are excluded from further analyses.

Table 4 also shows the consistencies for each of the three performance outcomes. Consistency refers to the degree to which the configuration is a subset of the outcome. For crisp sets, this would equal the percentage of firms in the configuration that have the high-performance outcome. In the case of fuzzy sets, consistency cannot be directly calculated as such a percentage, but the interpretation follows similar logic. For example, out of the 19 firms with high EO, few problems, high pivoting, and low perseverance in configuration EpVc, 89% think their performance is good, while 93% have a high ROA and 90% have good growth performance. The consistencies for the negated outcomes are also shown.

### Analysis of sufficiency – Boolean minimization

The analysis of sufficiency aims to identify the configurations that are sufficient for the outcome; i.e. they are consistent subsets of the outcome set (Greckhamer et al., 2018). In other words, when a particular configuration is sufficient, all cases within it exhibit the outcome. In the analysis of sufficiency, it is critical that the consistency is high enough, and the usual recommended threshold is 0.80 (Ragin, 2008). In large samples, it is common to observe contradictory configurations (i.e. it can be observed that cases within a single configuration exhibit a different outcome), which by definition lowers the consistencies (Greckhamer et al., 2013). The Boolean minimization was conducted using the Quine–McCluskey algorithm (Ragin, 2017, p. 37). In line with recommendations by Greckhamer et al. (2018), we analysed the configurations for the presence and the absence of the outcome separately. Table 5 shows the results of the analysis of sufficiency for our subjective performance measure.

**Table 5:** Analysis of sufficiency

Conditions	Good performance			Poor performance			
	a	b	c	d	e	f^1^	g^1^
EO (E)	●	●			○	○	○
Problems (P)	○		○	○	●		●
Pivoting (V)		●	●	●	○	●	
Perseverance (C)	○	○		●		●	●
Consistency	0.88	0.84	0.84	0.82	0.84		
Raw coverage	0.50	0.44	0.50	0.34	0.44		
Unique coverage	0.11	0.04	0.10	0.10	0.20		
Solution consistency	0.81			0.80			
Solution coverage	0.64			0.54			

^1^Solution for poor subjective performance includes d, e, and either f or g

● means that condition is present, and ○ means that condition is absent

For the outcome of *subjective performance*, six configurations marked with an asterisk in Table 4 meet the thresholds of frequency above three and consistency significantly above 0.80, thus qualifying for the Boolean minimization. The solution covers 64% of all firms with good performance. However, the consistency of the solution is only marginally good at the level of 0.81. The Boolean minimization results in three prime implicants. The first one (a) with a high coverage (0.50) has a high level of EO combined with few problems and no attempts to persevere by cutting or postponing costs. Configuration (a) implies that, in the absence of major problems, entrepreneurial firms should avoid the perseverance strategy during the pandemic. Configuration (b) indicates that regardless of the number of problems, a combination of high EO and pivoting strategy while avoiding perseverance would also lead to good performance. Finally, configuration (c) implies that if problems stemming from the crisis are minor, pivoting would lead to good subjective performance, regardless of EO or perseverance.

The subjective performance is consistently poor when the firm attempts to pivot and persevere at the same time, while problems are small (d), regardless of the level of EO. Another path leading to poor performance is the combination of non-entrepreneurial firms failing to pivot in the presence of major problems (e). Furthermore, in non-entrepreneurial firms, the combination of perseverance with either the pivoting strategy (f) or major problems (g) is a recipe for failure. The solution coverage (0.54) is somewhat lower than the coverage for the good performance solution, suggesting that SMEs facing an external crisis and suffering from poor performance are more heterogeneous than those performing well.

### Robustness tests

Robustness checks were run (1) by replacing the subjective performance outcome with the objectively measured outcomes, (2) by varying the frequency threshold, which was dropped to 1, and (3) by varying the fuzzy-set calibration method into a direct, partly theoretically driven one, as explained earlier in "Measure calibration" section.

The results of the first robustness check are shown in Appendix 3, Table 9. When firm performance is more objectively measured using *ROA*, we find a consistent (0.82) solution that covers 60% of high-profitability companies in the sample. The solution includes three prime implicants: h and i imply that perseverance strategy should be avoided when problems are small if the firm is either pivoting or has a high level of EO. The combination of EO and pivoting leads to good performance in the absence of perseverance regardless of the severity of problems (j). Interestingly, the configurations resulting consistently in high ROA are nearly identical to those explaining good subjective performance. The annual growth of sales consistently occurred in two prime implicants, and the solution coverage was satisfactory (0.57). Pivoting strategy implies growth during the pandemic when the firm has not experienced major problems or tried to persevere by cutting costs (k). In addition, when the firm has not faced many problems, a high EO combined with no perseverance results in growth (l). Both prime implicants, which are consistently associated with growth, were also present in explaining high profitability.

There is only one configuration leading to consistently low profitability (m), covering about 31% of the firms with low ROA. This is a situation in which a very entrepreneurial firm faces major problems but tries to cope only by persevering with no attempt to pivot. Likewise, there is one prime implicant for a large decline in sales (n); this occurs consistently for non-entrepreneurial firms trying to cope with severe problems by perseverance.

In the second robustness check, lowering the frequency cutoff to 1 improved the coverage of the good performance solutions, but at the same time, consistencies slightly decreased (see Appendix 3, Table 10). The results indicate that the solution is quite robust for all three performance indicators. The combination of high EO, only minor problems, and no perseverance strategy (Epc) appears in all solutions. Similarly, the absence of major problems combined with the pivoting strategy (pV) seems to result in high performance in all analyses. Regarding the configuration (EVc), the robustness analysis confirms the idea that pivoting should not be combined with perseverance, but unlike the main analysis, the robustness analysis solutions indicate that this applies to both entrepreneurial and non-entrepreneurial firms.

In the second robustness test for poor performance, the consistencies remained very similar to the main analysis, but the coverages increased. However, the coverages for poor performance are clearly lower than those for good performance. The existence of major problems combined with a lack of EO seems to be a common configuration leading to poor performance, especially if the firm tries to cope with perseverance.

In the third robustness check, applying the direct calibration of measures further supported the findings for good performance outcomes (see Appendix 3, Table 11). While the solutions are not identical to the ones described previously, the configurations Epc, EpV, and pV appear consistently across all performance measures. In terms of poor performance, the finding is robust for the subjective evaluation of performance, whereas there is no consistent solution for poor ROA and sales decline if the variables are calibrated using theoretically driven thresholds.

## Discussion and conclusions

### Summary of results

The main goal of this study was to identify configurations of EO, level of problems caused by an external crisis, and coping strategies that are consistently linked with good and poor firm performance during the crisis. With this aim, a dataset consisting of survey answers from 201 owner-managers of Finnish SMEs measuring the level of EO, COVID-related changes in the firms’ operating environment, firms’ response strategies, and subjective firm performance combined with official financial statement figures was built. Due to the configurational nature of the developed theoretical model, the fsQCA methodology was applied in the analysis.

Our results indicated that Finnish SMEs, on average, were not very severely hit by the pandemic during its first year. Most respondents perceived their financial performance to be on a good level, and this was also evident from the financial reports. As a response to the crisis, it was more common to apply pivoting than perseverance strategies. EO was positively related to pivoting, growth, and subjective performance but not related to profitability. Firms that had faced more problems tended to apply the perseverance strategy, and their financial performance was weaker.

The most frequent configurations among the SMEs were the extremes: (a) the firm faced a lot of problems, had a high EO, and applied both coping strategies, and (b) the problems were minor, EO was moderate, and no coping strategies were employed. However, these configurations were heterogeneous (inconsistent) in terms of financial performance.

The reduced sets that consistently implied good performance in our main analysis and robustness checks were configurations in which firms facing minor problems either pivoted or had a high EO while avoiding the perseverance strategy. In addition, regardless of the problems faced, highly entrepreneurial firms applying the pivoting strategy and avoiding the perseverance strategy enjoyed high subjective performance and ROA, but this configuration was not related to growth.

We also obtained the combinations of factors that lead to poor performance: non-entrepreneurial firms that faced major problems consistently scored poorly on all performance measures, regardless of the coping strategies employed. Furthermore, the application of both coping strategies by non-entrepreneurial firms turned into poor performance regardless of the problems faced by the firm.

### Implications

We believe that the contribution and importance of this study emerge from developing measures for crisis management and introducing the interplay of a generic strategic orientation, i.e. EO and concrete crisis management actions. Our paper has several contributions to the existing literature. First, the contribution stems from the fact that our paper is one of the first quantitative studies in the management field on the path started by Wentzel et al. (2020) and later followed and extended by Kraus et al. (2020) as we study the role of response strategies in crisis. Our quantitative results support the qualitative findings of Kraus et al. (2020) as we show that firms are combining different response strategies rather than solely relying on one single strategy while defending their business from exogenous shock. Furthermore, as an important finding, we are able to point out that every response strategy cannot be successfully combined with each other. This also relates to the ambidexterity literature, highlighting the difficulty in finding the strategic balance in a crisis situation.

Second, we were able to demonstrate that entrepreneurially oriented firms have better performance in challenging circumstances than their less entrepreneurial counterparts. As Covin et al. (2020) point out, the relationship between EO and performance might be more complex than previously assumed and hence studies that focus on the interplay between EO postures and firm-level strategies are necessary. In addition, we argue that EO as a strategic orientation is difficult to change, meaning that coping strategies should be aligned with it. Therefore, our paper contributes to the EO literature by responding to the call for more research with a focus on the interaction between EO postures and firms’ strategy choices as we manage to empirically confirm the argument made by Linton and Kask (2017) that combining a firm's EO posture correctly to its strategy might affect performance positively. Moreover, besides just confirming the positive role of EO, our findings also show the dark side of EO as we find configurations where EO results in poor performance. A minor contribution to EO literature also comes from the measures at our disposal; as Huang et al. (2022) point out the importance and need to utilize multiple measures for performance in EO studies, we use objective sales growth and profitability in addition to subjective performance measures, allowing us to examine the outcome variable from multiple views. Our study also makes a general contribution to SME literature by showing how different types of coping strategies are powerful and effective in different intensities of the crisis experienced by the firms.

The results of our work are also useful for entrepreneurs. For SMEs, it is important to recognize that during the crises it is important to ensure liquidity but also innovate and take advantage of short-term opportunities and develop further the existing business model. The difficult times can also be the path to developing new business models and pivoting them. We suggest that government support and policies have an important role in guiding firms to successful pivots. For SMEs, it is also important to understand that financial support by the government is not intended to keep existing business ongoing, it is an extra resource for innovation, and it is intended to turn the current business into a new angle.

### Limitations and further research

Like all studies, our study has several limitations that could hopefully be overcome in future research. First, in QCA, the number of configurations exponentially increases with the number of conditions, making it difficult to include control variables. For the same reason, the crisis coping strategies in this study were examined on only two dimensions (pivoting as the offensive/active strategy and perseverance as the defensive/passive strategy). We acknowledge that a more refined dimensionality of coping strategies could offer a more nuanced picture of how firms effectively deal with crises. For example, the separation of perseverance to maintain the status quo and retrenchment, as theorized by Wentzel et al. (2020) and qualitatively discovered by Kraus et al. (2020), could be a fruitful way to empirically examine coping strategies in future studies. However, in our sample, the items related to retrenchment correlated strongly with the perseverance items, and thus we are confident that our parsimonious model captures the main aspects of coping strategies while maintaining the simplicity of interpretation.

Second, the survey data were collected in 2020, and the latest financial reports are from the year 2020. This means that our study is not able to capture the long-term impacts of the crisis itself or the coping strategies applied. The restrictions caused by the pandemic were mostly released in the spring of 2022, when another external crisis – the Ukraine conflict – caused further restrictions on Finnish companies, especially those that had business relationships with Russia. The specific effects of these two partly overlapping different types of crises on different types of firms may vary, but as such the crisis response strategies and EO should be applicable across different contexts. In future studies, it would be interesting to find out how pervasive the coping strategies are in a lengthened crisis or in a series of separate crises. A longitudinal study would also enable empirical testing of the conceptual ideas about the effectiveness of pivoting and perseverance strategies in the short vs. long term (Wentzel et al., 2020).

Third, the data was collected in only one country, Finland. The main constructs of interests (effects of crisis on SMEs, crisis response strategies, EO and financial performance) should be applicable to other similar developed countries. Obviously, the situation might be very different in developing countries, where the lack of institutional support would increase the effects of the crisis on firms and also limit the possibilities for e.g. perseverance coping strategy. Future studies in different types of countries could shed more light on the contextual contingencies between the coping strategies and firm performance in crisis situations.

Fourth, the measurement of problems and coping strategies was not based on fully validated scales. The measures were inspired by previous studies but were significantly adapted to fit the context of Finnish SMEs in the COVID-19 setting. However, the applied scales can provide a basis for future research and validation in different samples and different types of crises. Finally, the generalizability of our results outside of Finland is limited. The timing and spreading of the pandemic differed a lot between countries, and governments took different approaches in dealing with it. For example, the lockdowns would impact the intensity of the problems faced by SMEs in different ways across countries, and the governments’ varying support funding could support different kinds of coping strategies.

Finally, this study only focused on SMEs, as they have become an increasingly important component of economic development. The role of SMEs as major job suppliers, innovators, and sources of growth in free market economies have long been recognized. However, this global economic crisis has also been severe for large enterprises, and their response strategy choices might be different due to their more extensive resources.

## Appendices

### Appendix 1. Measurement scales

#### Subjective firm performance

The scale is composed of the mean of the following three items:

*Please evaluate your performance compared to that of your main competitors in the same industry. Response scale 1 = a lot worse than competitors, 5 = a lot better than competitors.*

- Profitability
- Growth

- Ability to survive through the crisis

#### Problems

The formative scale is composed of the mean of the following nine items:

*What kinds of issues have you faced during the crisis? Scale 1 = totally disagree, 5 = totally agree.*

- The crisis has decreased our sales significantly.
- The crisis has decreased our profitability significantly.
- The crisis makes our operations harder overall.
- Customers have cancelled their orders.
- The amount of work has decreased significantly.
- It has become harder to get financing.
- Our credit losses have increased.
- Our suppliers have tightened their payment terms.
- Our suppliers have not been able to deliver.

#### Entrepreneurial orientation

Table 6

**Table 6:** Entrepreneurial orientation – measurement items and factor analysis results

Items *(Please evaluate your firm’s typical behaviour in a normal situation. Scale 1* = *totally disagree, 5* = *totally agree)*	Factor 1 loading	Factor 2 loading	Uniqueness
Continuous renewal and innovation are important for our company	0.816		0.318
We invest heavily in developing new products, services, and business practices	0.790		0.292
In our company, new ideas come up all the time	0.837		0.269
We aim at being at the forefront of development in our business sector	0.700		0.451
Lately, we have launched many new products/services	0.674		0.532
Our company often acts before the competitors do	0.684		0.530
In uncertain situations, we are not afraid to take substantial risks	0.622	− 0.455	0.405
Bold action is necessary to achieve our company’s objectives	0.715		0.454
We prefer the cautious line of action even if some opportunity might be lost that way (reversed)		0.764	0.285
Eigenvalue	4.434	1.029	
% of variance	49.27	11.43	

Principal component analysis with Varimax rotation. Blanks represent abs(loading) < 0.40

#### Crisis coping strategies

Table 7

**Table 7:** Crisis coping strategies – measurement items and factor analysis results

Items *(What have you done in response to the crisis? Scale 1* = *totally disagree, 5* = *totally agree)*	Perseverance factor loading	Pivoting factor loading	Uniqueness	KMO
We have dismissed personnel	0.56		0.69	0.82
We have laid off personnel	0.65		0.56	0.85
We have needed extra financing to secure our operations	0.64		0.55	0.89
We have cancelled our investments	0.78		0.38	0.84
We have delayed our investments	0.77		0.40	0.83
We have evaluated our solvency more carefully before paying dividends	0.57		0.52	0.88
We have cancelled our decision to pay dividends	0.74		0.45	0.85
We have postponed our decision about dividends	0.77		0.41	0.82
We have considered exit	0.55		0.70	0.83
Our managers have worked for months without pay or with reduced pay	0.73		0.45	0.91
We have developed new services or products		0.76	0.41	0.77
We have taken up new distribution channels, such as e-commerce		0.46	0.77	0.68
We have targeted marketing to new customer segments		0.77	0.37	0.75
We gave got new customers from new segments		0.74	0.44	0.71
We have learned new modes of operation, which will remain		0.77	0.41	0.78
Eigenvalue	5.09	2.40		
Cumulative % of variance	0.34	0.50		
Cronbach alpha	0.87	0.75		

Principal component analysis with Varimax rotation. Blanks represent abs(loading) < 0.40. Overall KMO = 0.83

### Appendix 2

**Table 8:** Calibration thresholds applied in robustness tests

Outcomes	Full member (90th percentile)	Cross-over (median)	Non-member (10th percentile)
Subj.perf	4.7	3.7	3.0
ROA	10	5	0
Growth%	0	−10	−20
Conditions			
EO	4.3	3.4	2.5
Problems	3.7	2.0	1.25
Pivoting	4.0	3.0	1.4
Perseverance	3.5	1.6	1.0

### Appendix 3. Robustness tests

Tables 9, 10, and 11

**Table 9:** Results of robustness check 1 (objective performance as outcomes)

	High ROA			Sales growth		Low ROA	Sales decline
Conditions	h	i	j	k	l	m	n
EO (E)		●	●		●	●	○
Problems (P)	○	○		○	○	●	●
Pivoting (V)	●		●	●		○	
Perseverance (C)	○	○	○	○	○	●	●
Consistency	0.91	0.85	0.88	0.86	0.85	0.87	0.87
Raw coverage	0.48	0.50	0.44	0.47	0.51	0.31	0.45
Unique coverage	0.07	0.09	0.03	0.06	0.10	0.31	0.45
Solution consistency	0.82			0.82		0.87	0.87
Solution coverage	0.60			0.57		0.31	0.45

● means that condition is present, and ○ means that condition is absent

**Table 10:** Results of robustness check 2 (frequency cutoff = 1)

Subj.perf. good		Total cov	Sol. cons	Subj.perf. poor		Total cov	Sol. cons
		0.67	0.78			0.58	0.80
Set	Raw cov	Unique cov	Cons	Set	Raw cov	Unique cov	Cons
epC	0.32	0.02	0.83	eC	0.51	0.05	0.82
Epc	0.50	0.09	0.88	eP	0.53	0.07	0.82
pV	0.50	0.02	0.84				
Vc	0.50	0.04	0.80				
ROA good		Total cov	Sol. cons	ROA poor		Total cov	Sol. cons
		0.69	0.79			0.42	0.86
Set	Raw cov	Unique cov	Cons	Set	Raw cov	Unique cov	Cons
ePc	0.39	0.04	0.89	ePvc	0.32	0.11	0.86
Epc	0.50	0.07	0.85	EPvC	0.31	0.10	0.87
pV	0.53	0.04	0.88				
Vc	0.53	0.02	0.85				
Sales growth		Total cov	Sol. cons	Sales decline		Total cov	Sol. cons
		0.61	0.81			0.56	0.86
Set	Raw cov	Unique cov	Cons	Set	Raw cov	Unique cov	Cons
Epc	0.51	0.10	0.85	eVC	0.39	0.02	0.90
pV	0.51	0.10	0.84	eP	0.53	0.17	0.86

**Table 11:** Results of robustness check 3 (direct calibration)

Subj.perf. good		Total cov	Sol. cons	Subj.perf. poor		Total cov	Sol. cons
		0.64	0.81			0.51	0.82
Set	Raw cov	Unique cov	Cons	Set	Raw cov	Unique cov	Cons
epvC	0.31	0.04	0.85	eP	0.51	0.51	0.82
ePvc	0.28	0.03	0.87				
pVc	0.30	0.02	0.87				
Epc	0.46	0.09	0.89				
EpV	0.30	0.03	0.88				
Evc	0.30	0.02	0.85				
ROA good		Total cov	Sol. cons	ROA poor		Total cov	Sol. cons
		0.28	0.90				
Set	Raw cov	Unique cov	Cons	Set	Raw cov	Unique cov	Cons
pV	0.28	0.28	0.90	none			
Sales growth		Total cov	Sol. cons	Sales decline		Total cov	Sol. cons
		0.22	0.90				
Set	Raw cov	Unique cov	Cons	Set	Raw cov	Unique cov	Cons
EpV	0.22	0.22	0.90	none			
